# Preparation of a Cage-Type Polyglycolic Acid/Collagen Nanofiber Blend with Improved Surface Wettability and Handling Properties for Potential Biomedical Applications

**DOI:** 10.3390/polym13203458

**Published:** 2021-10-09

**Authors:** Sofia El-Ghazali, Hisatoshi Kobayashi, Muzamil Khatri, Duy-Nam Phan, Zeeshan Khatri, Sheeraz Khan Mahar, Shunichi Kobayashi, Ick-Soo Kim

**Affiliations:** 1 Faculty of Science and Technology, Division of Biomedical Engineering, Department of Biomedical Engineering, Shinshu University, Tokida 3-15-1, Ueda 386-8567, Nagano Prefecture, Japan; elghazalisofia@gmail.com; 2 International Center for Materials Nanoarchitectonics (MANA), National Institute for Materials Science (NIMS), Tsukuba 305-0044, Ibaraki Prefecture, Japan; kobyying@gmail.com; 3 Nano Fusion Technology Research Group, Division of Frontier Fibers, Institute for Fiber Engineering (IFES), Interdisciplinary Cluster for Cutting Edge Research (ICCER), Shinshu University, Tokida 3-15-1, Ueda 386-8567, Nagano Prefecture, Japan; muzamilkhatri@gmail.com; 4 School of Textile-Leather and Fashion, Hanoi University of Science and Technology, No 1 Dai Co Viet Street, Hanoi City 10000, Vietnam; nam.phanduy@hust.edu.vn; 5 Center of Excellence in Nanotechnology and Materials, Mehran University of Engineering and Technology, Jamshoro 76060, Sindh, Pakistan; zeeshan.khatri@faculty.muet.edu.pk (Z.K.); 15mt96@students.muet.edu.pk (S.K.M.); 6 Department of Chemistry and Materials, Shinshu University, Tokida 3-15-1, Ueda 386-8567, Nagano Prefecture, Japan

**Keywords:** electrospinning, nanofiber blend, surface modification, ozonation, plasma treatment, cage-type collector, handling properties

## Abstract

Electrospun biobased polymeric nanofiber blends are widely used as biomaterials for different applications, such as tissue engineering and cell adhesion; however, their surface wettability and handling require further improvements for their practical utilization in the assistance of surgical operations. Therefore, Polyglycolic acid (PGA) and collagen-based nanofibers with three different ratios (40:60, 50:50 and 60:40) were prepared using the electrospinning method, and their surface wettability was improved using ozonation and plasma (nitrogen) treatment. The effect on the wettability and the morphology of pristine and blended PGA and collagen nanofibers was assessed using the WCA test and SEM, respectively. It was observed that PGA/collagen with the ratio 60:40 was the optimal blend, which resulted in nanofibers with easy handling and bead-free morphology that could maintain their structural integrity even after the surface treatments, imparting hydrophilicity on the surface, which can be advantageous for cell adhesion applications. Additionally, a cage-type collector was used during the electrospinning process to provide better handling properties to (PGA/collagen 60:40) blend. The resultant nanofiber mat was then incorporated with activated poly (α,β-malic acid) to improve its surface hydrophilicity. The chemical composition of PGA/collagen 60:40 was assessed using FTIR spectroscopy, supported by Raman spectroscopy.

## 1. Introduction

Recently, many scientists have been exploring the biomedical properties of different polymeric materials, and the preparation of such materials with multiple desired characteristics has remained a challenging task [1,2,3,4].

In the last few years, there has been a growing interest in Polyglycolic acid (PGA), collagen and their blended compositions incorporated with different functional guest materials for various targeted applications [5,6,7]. Only a few reports could be found regarding electrospun PGA, collagen nanostructures and their blends being incorporated with external functional guest materials to improve surface properties viz. wettability and morphology, and this remains a big research gap [8,9,10,11].

PGA-based copolymers have been considered as green polymers [1,6] with good biodegradability [6] and mechanical properties [12], a high heat distortion temperature [13], good biocompatibility [14], cell viability and very low solubility in organic solvents [15,16]. In the form of nanofibers, PGA-based copolymers have exclusive scaffolding properties for tissue regeneration, comparatively faster than polyesters, polycaprolactone (PCL) and polybutylene adipate-co-terephthalate (PBAT), which reveals a higher level of suitability of PGA for biomedical applications [6,17].

In parallel, collagen has also been utilized for several biomedical applications because of its biological characteristics, such as biodegradability, biocompatibility, low immunogenicity, easy processing and hydrophilicity [7].

It is well known that mammals contain an abundant amount of collagen in their structural protein, which has been utilized in various forms, such as membrane, hydrogel, powder, sponge and fibers [9,10,11]. Functionally, collagens are complex and structurally diverse. The literature confirms that collagen can perform well even when composited in the shape of fibers and utilized for potential biomedical applications in parallel to good physicochemical and mechanical properties [15,16].

Furthermore, collagen constitutes a fundamental part of the skin, tendons, sclera, ligaments, cartilage, cornea and bones [9,15]. The bulk presence of collagen in animals shows its biocompatibility, and materials based on collagen may be utilized to assist surgical operations [9,10,11,12,13,14,15]. 

Thusly, both PGA and collagen are considered as biomaterials and have been utilized for applications where self-supramolecular assembly, an extracellular matrix structure (ECM) interface and good bio-induction are required. However, these biomaterials may lose their originality when blended with external substrates to impart required characteristics such as tunable wettability. To prevent the latter issue, optimization using external surface treatments viz. plasma/ozonation or the incorporation of functional materials is needed [15,16,17].

PGA/collagen nanofiber-based scaffolds have very good cell adhesion and cell growth properties but are not sufficient for other practical applications, such as bleeding control during surgeries [18,19,20,21,22,23,24,25], material flexibility and easy handling during surgeries [19,20,21,22]. These critical issues during surgeries can be addressed by modifying the surface and handling properties [22,23,24,25,26,27,28,29,30].

Therefore, a blended composition is necessary to impart suitable surface properties onto the optimized nanofibers in order to achieve an ease in handling and the ability to control severe bleeding during surgical operations [24,25,27].

Changes in the surface properties of electrospun biomaterials, such as texture, wettability and present functional groups [3,26], can be altered either via surface treatments (plasma/ozonation) or by incorporating functional guest molecules which may introduce required chemical groups on the surface [31,32,33,34,35,36].

Nanofibers possess a high surface area, a very fine fiber diameter and have been utilized for multipurpose applications, including tissue engineering [37,38,39,40,41], smart apparel and environmental applications due to the ease of their production [42,43,44,45,46,47,48]. Their high breathability, light weight, biocompatibility and three-dimensional morphology make them a porous matrix which resembles the native ECM [38,39,40].

Nanofiber-based composites, due to the considerable ease with which they can be handled and their optimized surface properties, can be potentially utilized for assisting biomedical surgical operations [49,50]. Ultimately, the optimal wettability of polymeric materials has been considered as an important parameter for cell culture and cell adhesion [51,52,53,54,55,56,57]. Therefore, a bio-based cage-type nanofiber blend PGA/collagen was prepared using a cage-type collector during electrospinning, and the effect of ozonation and plasma treatments was assessed. Additionally, two concentrations of poly (α, β-malic acid) (SuPMA) were incorporated into the cage-type blend [58], and the wettability was checked using the WCA test, supported by Raman and FTIR spectroscopy.

## 2. Experimental Section

### 2.1. Materials

PGA, lyophilized type I collagen (derived from porcine tissue) and chemical grade 1,1,1,3,3,3 hexafluoro-2-propanol (HFIP) were purchased from Sigma-Aldrich, Ltd., Osaka, Japan. SuPMA was synthesized as per the method used in a previous study [58].

#### Preparation of PGA and Collagen and Cage-Type Nanofiber Blend via Electrospinning

**Figure 1:**
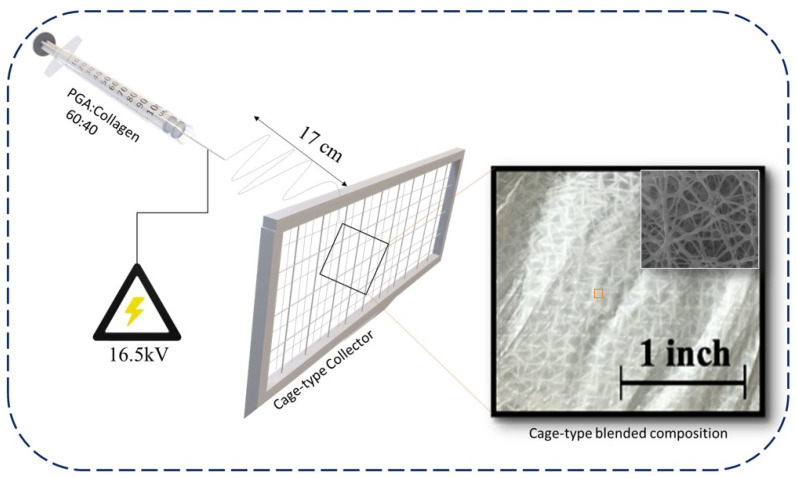
Preparation of cage-type PGA/collagen (60:40) nanofiber blend.

Both PGA and collagen polymer solutions were separately prepared with 10% (*w*/*v*) polymer concentration in HFIP. PGA and collagen were dissolved at 70 °C and 4 °C for 80 h and 24 h, respectively. Collagen and PGA were blended at different ratios (60:40, 50:50 and 40:60 (*v*/*v*)). The blends of PGA/collagen were poured in a 10 mL syringe affixed with a 22-gauge stainless needle, and the feed rate of the syringe pump (780100J, KD Science, Holliston, MA, USA) was set at 0.4–0.7 mL/h. A high-voltage (16.5–18.2) kV was supplied to the needle using an HSP-30K-2 (Nippon Stabilizer Industry Co. Ltd., Osaka, Japan), keeping the bead-free nanofibers production in consideration, for electrospinning for 4.5 h at room temperature with humidity maintained at 30 ± 6% by the perfusion of nitrogen. A cage-type metallic collector with pore size 2 × 2 mm^2^ and a plane metallic plate were used for the collection of nanofibers during electrospinning. All blended compositions of nanofiber layers were peeled and air dried before further assessment [32]. Figure 1 shows a demonstration of electrospinning of a cage-type nanofibers web; it further reveals the photographic image of the cage-type nanofibers with an inset SEM image, revealing its smooth and bead-free morphology.

## 3. Characterization

### 3.1. Scanning Electron Microscopy (SEM)

Neat PGA, neat collagen and all treated (using plasma and ozonation) and untreated blended compositions were examined under SEM (JEOL-5600LV, Japan) at an accelerating voltage of 20 kV. A JEOL-JED2200 (Japan) was used to carry out EDS and the samples were coated with tungsten and platinum for the morphological observations using Elionics-E101. The average diameter histogram of all nanofiber samples was analyzed using SEM JEOL-5600LV-based software.

### 3.2. Fourier Transform Infrared (FTIR) Spectroscopy

All nanofiber samples were assessed using IR Prestige-21, Shimadzu (Kyoto, Japan) in ATR-FTIR mode at an adsorption wavelength between 700 cm^−1^ and 4000 cm^−1^ at 25 °C.

### 3.3. Water Contact Angle (WCA) Test

The WCA was measured using the static angle by a drop testing method, keeping the drop size at 1 μL. The average taken from 15 measurements was considered from 1 to 300 s. FACE model CA-VP, Kyowa interface science, Japan was used to check the WCA of neat PGA, collagen, their blended compositions, plasma-treated PGA, collagen, their blended compositions, ozone-treated PGA, collagen, their blended compositions and the neat cage-type blend with and without the incorporation of SuPMA.

### 3.4. Raman Spectroscopy

The Raman spectra of the cage-type PGA/collagen was checked using Raman Touch-VIS-NIR, Nano photon, and the results were recorded at room temperature in the range between 800 cm^−1^ and 4000 cm^−1^.

## 4. Results and Discussion

### 4.1. Morphology of PGA, Collagen, and Their Blend Nanofibers

**Figure 2:**
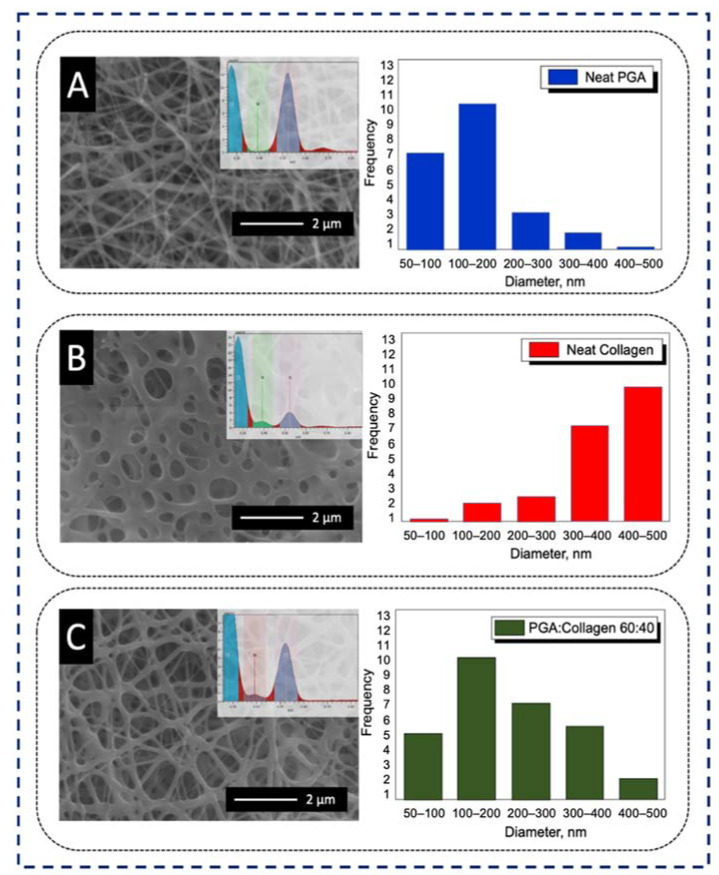
SEM images, EDS and diameter distributions histogram of (**A**) neat PGA, (**B**) neat collagen, (**C**) PGA/collagen (60:40).

Electrospinning of neat PGA with a concentration of 10% showed a bead-free morphology with the random deposition of fine nanofibers with an average diameter of 180 ± 30 nm, as shown in Figure 2A. Neat collagen showed semi-degraded and thick nanofibers with an average diameter of 50 ± 40 nm, as revealed in Figure 2B. The PGA/collagen nanofibers with an optimized ratio of 60:40 showed a bead-free, smoother morphology and regular deposition as compared to neat PGA and neat collagen nanofibers, as revealed in Figure 2C. An average diameter distribution of 300 ± 20 nm was observed for the blended composition, which is between the average nanofiber diameter of neat PGA and neat collagen. In addition, SEM-EDS confirmed the presence of both PGA and collagen in the resultant blend, and an abundancy of oxygen from PGA and nitrogen from collagen can clearly be seen in the PGA/collagen 60:40 blend, as per the inset EDS graphs given in Figure 2A–C, respectively.

### 4.2. Effect of Ozonation and Plasma Treatment on the Morphology of PGA/Collagen Blends

**Figure 3:**
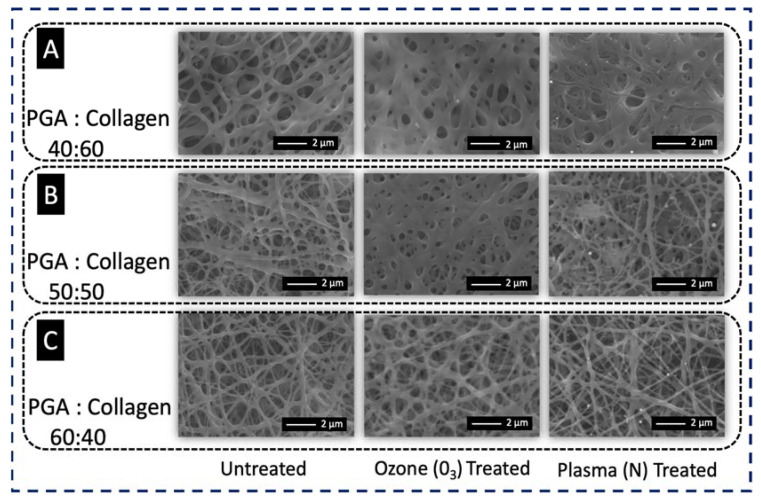
SEM images of untreated, ozone (O_3_)-treated, and plasma (N)-treated (**A**) PGA/collagen nanofibers with ratio 40:60, (**B**) PGA/collagen nanofibers with ratio 50:50 and (**C**) PGA/collagen nanofibers with ratio 60:40.

The choice of PGA/collagen 60:40 as the optimized blend was based on the comparison of the morphology of three ratios of PGA/collagen blends after ozonation (O_3_) and plasma (nitrogen) treatment. Ozonation and plasma treatments impart respective functional groups on the surface of suitable substrate. To treat surfaces, a constant time slot was fixed individually for ozonation (2 min) and plasma treatment (5 s) on each blended composition of PGA and collagen. Figure 3A shows thick nanofibers in PGA/collagen 40:60; during ozonation, the fibers were degraded at a certain level, and after plasma treatment, the fibers were semi-stable with a slight degradation. Comparatively, finer fibers in PGA/collagen 50:50 were observed, as shown in Figure 3B; this blend was slightly less degraded than the PGA/collagen 40:60. A root-shaped morphology was observed due to the breakage by plasma treatment’s pressure on the surface of PGA/collagen 50:50 nanofibers, and the nanofibers became comparatively thinner than the untreated PGA/collagen 50:50. Figure 3C reveals a slight degradation of PGA/collagen 60:40 on ozonation with fine and stable nanofiber morphology, whereas plasma treatment did not show any negative influence in the blended composition of PGA/collagen 60:40. In parallel to the smooth, regular and stable morphology of the PGA/collagen 60:40 blend, very good handling properties were observed. Thus, PGA/collagen 60:40 can be considered for its optimization and utilization in surgical operations.

### 4.3. Physico-Chemical Properties of PGA, Collagen, and Their Blend Nanofibers

**Figure 4:**
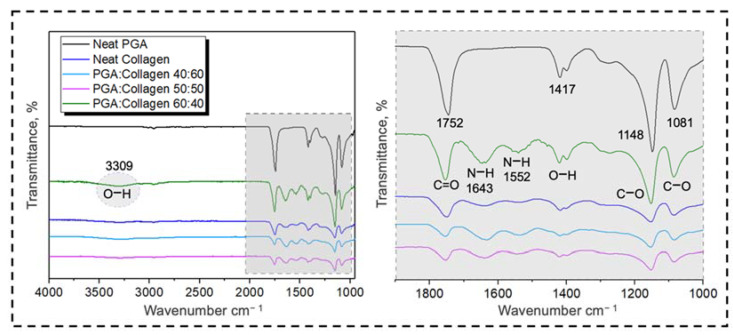
FTIR spectrum of neat PGA, neat collagen and their blended compositions.

Neat PGA, neat collagen and three different blends were assessed using FTIR spectroscopy, as shown in Figure 4. Neat PGA shows two big stretching peaks of C–O and C=O at 1148 cm^−1^ and 1752 cm^−1^, respectively, and one small peak at 1081 cm^−1^, confirming the bulk presence of C–O groups. The FTIR spectrum of PGA shows a bending peak at 1417 cm^−1^, revealing OH presence within the chemical structure of neat PGA nanofibers, whereas it does not show any peak of the OH group in the range near 3300 cm^−1^. A small peak at 710 cm^−1^ shows the presence of CH within neat PGA nanofibers.

On the other hand, neat collagen showed a raising peak at 3309 cm^−1^, confirming the presence of an OH group on its surface, and a small bend at 1417 cm^−1^ represents OH groups within the chemical composition of collagen nanofibers. The FTIR spectrum further shows NH at 1643 cm^−1^ and also demonstrates the presence of NH and OH at small peaks between 1400 cm^−1^ and 1600 cm^−1^. Similar to neat PGA, neat collagen also has stretching peaks at 1081 cm^−1^, and at 1148 cm^−1^, C–O is revealed to be present in its chemical structure. Neat PGA has more intensive peaks due to the bulk presence of chemical groups on its surface; therefore, PGA/collagen 40:60 did not show any intensive peaks at any point of the FTIR spectrum; even in the blends of PGA/collagen 50:50, some small peaks slightly appear, confirming the blending of PGA and collagen, while in the case of PGA/collagen 60:40, the peaks became intensive with clear stretching and bending, resulting in more visible OH groups on the surface of the blended composition compared to neat collagen, as per the given FTIR results. The FTIR spectra of blended PGA/collagen (60:40) shows a higher number of NH, C–O and N–O groups compared to the groups present in other PGA/collagen blends. Bulk functional groups on the blend surface have a direct impact on the wettability of polymeric fibers, which, according to the literature, may offer very good cell culture and adhesion properties compared to those with low surface wettability [52,54,55].

### 4.4. Effect on Wettability of Nanofibers from Neat and Blends of PGA and Collagen

#### 4.4.1. Ozonation and Plasma Treatment Effect on the Wettability of Nanofibers

**Figure 5:**
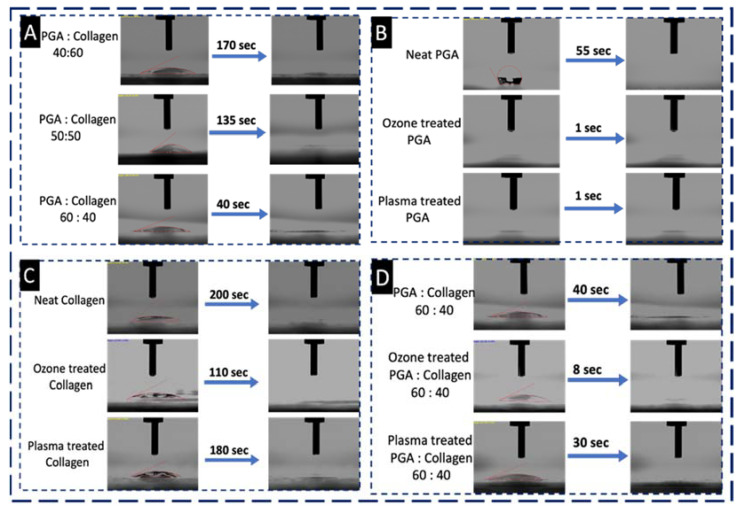
WCA test of (**A**) PGA/collagen nanofibers with blend ratios 40:60, 50:50 and 60:40, WCA of untreated, Ozone (O_3_) treated, and Plasma (N) treated (**B**) Neat PGA, (**C**) Neat collagen and (**D**) PGA/collagen 60:40.

The effects of ozonation and plasma treatments on the wettability of neat and blended compositions of PGA and collagen nanofibers were determined using a WCA test; FTIR results revealed the bulk presence of OH and NH groups on the surface of PGA/collagen with a ratio of 60:40, which was an indication of some change in the wettability on its surface. Figure 5A shows the water contact angle (WCA) test of PGA/collagen nanofibers with the blend ratios of 40:60, 50:50 and 60:40 and with water contact angles between 20° and 25°. The PGA/collagen 40:60 took 170 s to completely absorb the droplet on its surface suggesting its hydrophobic behavior. Decreasing the collagen polymer concentration to 50% into the blended composition resulted decreased hydrophobicity, which took 135 s to completely absorb the droplet as compared to the blend containing 60% of collagen. FTIR spectroscopy did not show any significant difference in the chemical structure of PGA/collagen with ratios 40:60 and 50:50 due to small difference in the composition blends, only the change in wettability showed the difference in blended composition which was due to more PGA content.

WCA of PGA/collagen 60:40 demonstrated only 40 s to completely absorb the water droplet on its surface where the contact angle was 20°, which is considered as hydrophilic, the reason to this was the presence of more NH and OH groups on its surface as confirmed by FTIR spectroscopy. WCA test revealed that the simple blending process of polymers (PGA and collagen) at room temperature in the presence of solvent (HFIP) can impart more functional groups on the surface of resultant PGA/collagen 60:40 nanofibers. Figure 5B shows the WCA of untreated, ozone treated and plasma treated PGA nanofibers. WCA of neat PGA nanofibers did not show very good hydrophilic properties with droplet absorption to 0° from 120° in 55 s, which is comparatively less hydrophilic than the optimized blend of PGA/collagen with ratio 60:40 followed by FTIR results. PGA on 2 min of ozonation, and 5 s of plasma treatment increased the wettability to super hydrophilic range with reduced droplet adsorption time to 1 s, whereas the initial contact angle was <10°. In Figure 5C, collagen also showed increased wettability with decreased time for complete droplet adsorption but still remained in the category of hydrophobic revealing 200 s, 100 s and 180 s for untreated, ozone treated and plasma treated collagen nanofibers having contact angles 36°, 23° and 23°, respectively, which individually may not be appropriate for good cell adhesion properties, because cell adhesion on biomaterials can be maximal when the hydrophilicity of the surface is optimal. The optimized blended composition PGA/collagen with ratio 60:40 was also treated using ozone and plasma, and assessed under WCA test as shown in Figure 5D. Neat PGA/collagen (60:40) took 40 s, ozone treated blend took 8 s, plasma treated sample took 30 s for complete droplet absorption on their surfaces, whereas, the contact angles were observed as 20°, 50°and 21°, respectively.

#### 4.4.2. Effect of SuPMA Incorporation and Cage-type Collector on Wettability of Blend Nanofibers

**Figure 6:**
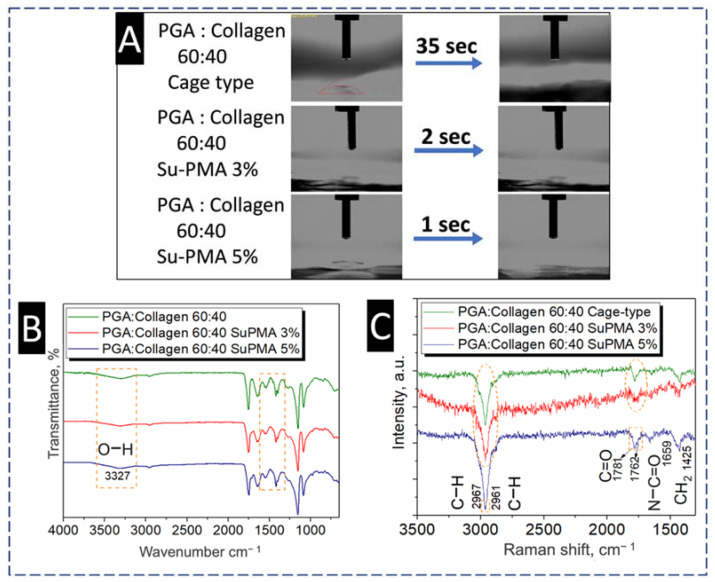
(**A**) WCA test of Cage-type PGA/collagen 40:60, (**B**) FTIR spectra of Cage-type PGA/collagen 40:60 with 3% SuPMA and cage-type PGA/collagen 40:60 with 5% SuPMA and (**C**) Raman spectra of Cage-type PGA/collagen 40:60 with 3% SuPMA and cage-type PGA/collagen 40:60 with 5% SuPMA.

WCA of (cage-type blend) PGA/collagen 60:40 is revealed in Figure 6A which interestingly showed more hydrophilicity compared to the smooth textured blend prepared on plan collector. Cage-type blend took only 35 s to make the contact angle reach 0° from 35.4°. Increased hydrophilicity is an obvious advantage of preparing a cage-type blend, which may be ultimately advantageous to cell adhesion properties. Additionally, the wettability of neat and SuPMA incorporated cage-type blend is compared in Figure 6A, the incorporation of SuPMA 3% and SuPMA 5% showed super hydrophilic behavior on the surface of cage-type blend which only took 2 s and ≤1 s, respectively for complete water droplet absorption on their surfaces where the contact angle was 10°. The reason behind the increased hydrophilicity is given in Figure 6B,C, showing FTIR and Raman spectroscopies, respectively to assess chemical groups available on the surface of cage-type blend, and to confirm the extent of incorporation of SuPMA. FTIR showed increasing cis peaks at 1400 cm^−1^ on the incorporation of SuPMA, which reveals more OH groups on the surface of 3% SuPMA incorporated cage-type blend with increased hydrophilicity compared to neat cage-type blend and comparatively less hydrophilicity than the sample incorporated with 5% SuPMA. The reason of increased hydrophilicity and extent of incorporation of SuPMA was further confirmed by Raman spectroscopy, which reveals a continued increase in CH groups with stretching peaks at 2961 cm^−1^ and 2967 cm^−1^. Raman spectrum also confirmed the presence of C=O, N–C=O and increased CH_2_ at 1781 cm^−1^, 1658 cm^−1^ and 1425 cm^−1^, respectively. As per previous reports, increased number of CH_2_ groups on polymeric fiber’s surfaces results in increased cell adhesion properties, which indicates the potential for cell culture applications of this optimized cage-type blend with 5% SuPMA content [59].

## 5. Conclusions

The cage-type blended composition of PGA/collagen nanofibers with ratio 60:40 was optimized to achieve the maximum wettability with good handling properties. Optimized PGA/collagen with the ratio 60:40 showed a smooth morphology, whereas other blended PGA/collagen compositions did not resist the ozonation and plasma treatments pressure. FTIR and SEM-EDS revealed the presence of both PGA and collagen in the resultant blended composition, whereas the optimized blend with a ratio of 60:40 showed more NH and OH chemical groups on its surface, which was the reason behind its increased wettability. The optimized blend of PGA/collagen nanofibers was individually treated under plasma and ozonation, which potentially enhanced the wettability on the surface of pristine nanofibers and blends of PGA and collagen nanofibers. The ozone-treated blend took 8 s for droplet absorption to be complete, whereas the plasma-treated sample took 30 s to reach the contact angle of 0°. A cage-type collector with pore size 2 × 2 mm^2^ for electrospinning was used as a collector to obtain robust nanofibers’ texture to impart handling flexibility, which interestingly increased the wettability of the optimized blend and took 5 s for water droplet absorption to be complete on the surface, as per WCA results. SuPMA with 5% of incorporation in the optimized blend resulted in super hydrophilicity with a contact angle of 10°, and complete droplet absorption within 1 s.

## Data Availability

The data presented in this study are available on request from the corresponding author. There is no public repository at our institution.
